# DendroBLAST: Approximate Phylogenetic Trees in the Absence of Multiple Sequence Alignments

**DOI:** 10.1371/journal.pone.0058537

**Published:** 2013-03-15

**Authors:** Steven Kelly, Philip K. Maini

**Affiliations:** 1 Department of Plant Sciences, University of Oxford, Oxford, United Kingdom; 2 Centre for Mathematical Biology, Mathematical Institute, University of Oxford, Oxford, United Kingdom; 3 Oxford Centre for Integrative Systems Biology, Department of Biochemistry, University of Oxford, Oxford, United Kingdom; University of Lausanne, Switzerland

## Abstract

The rapidly growing availability of genome information has created considerable demand for both fast and accurate phylogenetic inference algorithms. We present a novel method called DendroBLAST for reconstructing phylogenetic dendrograms/trees from protein sequences using BLAST. This method differs from other methods by incorporating a simple model of sequence evolution to test the effect of introducing sequence changes on the reliability of the bipartitions in the inferred tree. Using realistic simulated sequence data we demonstrate that this method produces phylogenetic trees that are more accurate than other commonly-used distance based methods though not as accurate as maximum likelihood methods from good quality multiple sequence alignments. In addition to tests on simulated data, we use DendroBLAST to generate input trees for a supertree reconstruction of the phylogeny of the Archaea. This independent analysis produces an approximate phylogeny of the Archaea that has both high precision and recall when compared to previously published analysis of the same dataset using conventional methods. Taken together these results demonstrate that approximate phylogenetic trees can be produced in the absence of multiple sequence alignments, and we propose that these trees will provide a platform for improving and informing downstream bioinformatic analysis. A web implementation of the DendroBLAST method is freely available for use at http://www.dendroblast.com/.

## Background

Inferring phylogenetic relationships between biological sequences is fundamental to nearly all aspects of contemporary biological research. In addition to the pivotal role these inferences play in progressing our understanding of the evolution and diversity of life, they also provide a platform on which algorithms that predict sequence structure and function can be developed. The majority of methods for inferring relationships between biological sequences are dependent on the construction of a multiple sequence alignment. The improvement of multiple sequence alignment methods over more than 20 years has resulted in the production of many different multiple sequence alignment methods whose performances on diverse data types can vary considerably [1], [2], [3]. Accurate multiple sequence alignment is of particular importance to phylogenetic analysis as in all alignment-based inference methods the alignment, once constructed is taken as given. Specifically, data which inhabit the same column in a multiple sequence alignment are assumed to be homologous. Therefore, errors in the multiple sequence alignment directly contribute to errors in phylogenetic trees [4], [5], [6], [7].

Given a multiple sequence alignment there are several methods for inferring phylogenies which vary in speed, accuracy and complexity. These methods range from those with fewer parameters ones such as neighbour-joining (for example QuickTree [8]), and minimum evolution (for example FastMe [9]) to those with more parameters such as maximum likelihood (for example RAxML [10]) and Bayesian (for example MrBayes [11]) methods. Several approaches have also been developed to simultaneously infer both multiple sequence alignment and phylogenetic trees such as SATe and STATalign [12], [13], [14]. Similarly other methods have been developed that use multiple rounds of multiple-sequence alignment, tree inference, data-partitioning and re-alignment to infer phylogenetic trees such as SATCHMO [15].

In addition to the multiple sequence alignment based methods above, other methods have also been developed to try and circumnavigate the multiple sequence alignment completely. Some of these methods utilise pairwise similarity scores between sequences for distance based hierarchical clustering. Popular amongst these algorithms are those that use BLAST scores or e-values such as ProtoMap [16], ProtoNet [16], CluSTr [17], CLUSS [18] and TribeMCL [19]. Programs have also been developed which adopt a hybrid approach. An example of this is COCO-CL [20] a method which infers hierarchical clusters from correlation between BLAST e-values by resampling sequences from a multiple sequence alignment, so this method is not multiple sequence alignment free. In addition to these methods word-frequency based methods have been developed to evaluate similarity between sequences [21], [22], [23] in the absence of pairwise alignments.

Here, we provide a novel BLAST-based hierarchical clustering algorithm called DendroBLAST which constructs phylogenetic dendrograms/trees from protein sequences using a combination of BLAST and minimum evolution clustering. The method uses the BLOSUM62 matrix of amino acid substitution to make small numbers of changes to the sequences to identify and discard weekly supported bipartitions in the tree. We propose that this method, which uses widely-used existing tools for sequence analysis, will provide a platform for improving and informing multiple aspects of downstream bioinformatic analysis including multiple sequence alignment generation and phylogenetic tree inference. A web implementation of the DendroBLAST method is freely available for use at http://www.dendroblast.com/.

## Methods

### Constructing DendroBLAST trees

To begin a BLAST database of a set of protein sequences is created and a matrix of all possible pairwise BLAST bit scores is computed using the blastp algorithm [24]. We define *s(A,B)* as the BLAST bit score for sequence B produced using sequence A as a query. In the case where no BLAST score was observed between two sequences the following rule was used to replace *s(A,B)* so that all sequences have non zero score values
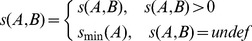
(1)where *s_min_(A)* is the minimum non-zero BLAST bit score value observed for sequence A when searching the entire dataset. BLAST bit scores will have different scales depending on the sequence and length of the query sequence. To account for this we compute a normalised bit score
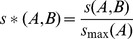
(2)where *s_max_(A)* is the maximum BLAST bit score observed for sequence A when searching the entire dataset. To take into account the dis-similarity in the pattern of BLAST hits produced using any two sequences we weight the normalised BLAST bit scores by the overlap in the number of sequences producing non-zero BLAST bit scores using each sequence as a query
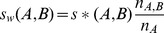
(3)where *n_A_* is the number of sequences producing non-zero BLAST bit scores identified in the dataset using sequence A as a query. *n_A,B_* is the number of sequences producing non-zero BLAST bit scores that are identified using both sequence A and B as queries, i.e. the overlap between the search results. Here sequences which produce more similar patterns of BLAST hits have higher weights so that two sequences which produce the highest BLAST bit score and have perfect overlap in the distribution of their BLAST hits will have a similarity score of 1. However, if there is no overlap between sequences producing BLAST hits then the inference procedure cannot be completed. As BLAST bit scores between any two sequences can be non-symmetric due to the properties of the query sequence we evaluate the similarity score between any two sequences as the mean of the weighted normalised BLAST bit scores
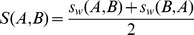
(4)such that *S(A,B)* = *S(B,A)* and *S(A,B)* is in the range (0,1]. Finally we convert this similarity score to a distance measure by taking the negation of the log of *S(A,B)*.
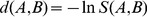
(5)where *d(A,B)* is the symmetric BLAST distance score (i.e. *d(A,B)* = *d(B,A)*) between sequence A and B. This similarity-to-distance score transform is similar to the BLAST bit score transform used by Lake *et al.* [25] except that in our method we provide additional steps to ensure the measure is symmetric and to take into account the similarity in the pattern of BLAST hits between sequences. Here, a BLAST distance score of 0 indicates that the pair of sequences is the highest scoring pair of sequences (including self-self pairs) and that there is perfect overlap between the sets of sequences identified by both query sequences. When all pairwise BLAST distance scores are computed a hierarchical cluster is inferred from this BLAST distance score matrix using the minimum evolution principle implemented in the FastMe algorithm [9]. In the case of the simulated sequence alignments BLAST similarity scores satisfy triangular inequality [26], however, in real sequence datasets non-metric constraints are imposed due to the modular nature of proteins and domains [27]. For example sequence A may contain two different domains, one of which it shares with sequence B and the other with sequence C. In this case sequence A will produce a non-zero BLAST score with sequence B and sequence C, but sequence B and C may fail to produce BLAST scores with each other.

### Testing the effect of sequence change on tree topology

To identify and remove poorly supported bipartitions, the tree inference procedure is repeated several times, each time introducing a small set of amino acid changes based on a probability distribution and the BLOSUM62 amino acid substitution rates (described below). A majority-rule consensus tree is then constructed from these replicate trees to eliminate the weakly supported bipartitions. To introduce these sequence changes the entire set of sequences is modified according to the following rule. The probability of replacement of each amino acid in each sequence is specified by a Gamma distribution distributed over an amino acid substitution matrix:
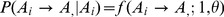
(6)where *P(A_i_→A_j_ | A_i_)* is the probability of substituting amino acid A_j_ for amino acid A_i_ given amino acid A_i_, *f(A_i_→A_j_; 1, θ)* is the probability density function of the Gamma distribution with shape 1 and scale θ. i.e.
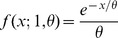
(7)The BLOSUM62 matrix was selected for use in the DendroBLAST procedure as this is the matrix used by the BLAST algorithm for evaluation of the BLAST bit scores. To facilitate the use of the BLOSUM62 matrix in the above schema, the integer values in the BLOSUM62 matrix were subject to the following transform. Each amino acid substitution value in the BLOSUM62 matrix was transformed so that the highest scoring substitution was set to 0 with all other substitutions having integer values greater than 0
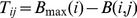
(8)Here, *B(i,j)* is the BLOSUM62 substitution value for replacing amino acid *i* with amino acid *j* and *B_max_(i)* is the maximum observed substitution value for amino acid *i*. For example, the BLOSUM62 score for substituting A with R is −1. The best scoring substitution for A is A itself which has a score of 4. When transformed so that the highest scoring substitution for A is assigned a value of 0 (and hence the most likely to be selected from the Gamma distribution) the value for substituting A with R becomes 5. However, in total 8 amino acids (R, Q, E, I, L, K, M, P) have the same score for replacing A. In these cases where a group of substitutions have the same score according to this normalised matrix the amino acid to be substituted was selected at random from this group. Therefore, the probability of replacing amino acid A with R in any given sequence can be evaluated as:
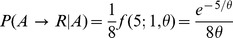
(9)where *P(A→R | A)* is the probability of substituting amino acid A with amino acid R given amino acid A. A Perl implementation of the DendroBLAST method is provided as File S1. The distribution of amino acid replacements is shown in Figure S1.

### Optimising the scale of the Gamma distribution

The optimal value for the scale of the Gamma distribution was determined using a randomly selected subset of 50 simulated protein sequence alignments from a previous analysis comprising 308 simulated multiple sequence alignments [28], [29]. These alignments were simulated on realistic tree topologies inferred from real sequence data derived from the COG database [28], [29], [30]. The alignments have realistic distributions of gaps and include rate variation across sites. For more detailed information on how these protein simulations were carried out see [28]. For each of the 50 randomly selected simulated sequence families, 100 replicates of the DendroBLAST amino acid replacement and tree inference procedure were performed. As DendroBLAST is a multiple sequence alignment free method these simulated alignments were parsed to remove all gap characters before being used for tree inference by DendroBLAST. In each case a majority-rule consensus tree was calculated from the 100 replicates using the python module dendropy [31]. The performance of DendroBLAST was evaluated as the mean Robinson-Foulds distance between the 50 inferred DendroBLAST trees and the 50 reference trees which were used to simulate the multiple sequence alignment. The Robinson-Foulds distance is the sum of the number of false positive and false negative bipartitions, where the false-positives (FP) are the set of bipartitions in the inferred tree not found in the reference tree and the false-negatives (FN) are the set of bipartitions present in the reference tree that are absent from the inferred tree.

To determine the optimal value for the scale of the Gamma distribution the inference procedure was repeated for a range of Gamma scale values between 0.2 and 5. 100 replicates were run without the amino acid replacement strategy and this value is shown at 0 and can be considered equivalent to BLAST clustering previously used [25]. For the range of Gamma scale values, the mean Robinson-Foulds distance of the test set was fit to a polynomial model using the polynomial curve fitting function (polyfit) in MATLAB (R2010b). The optimal value for the scale of the Gamma distribution was found as the minimum value of the fitted function over the interval interrogated (Figure S1). The Robinson-Foulds distance score was chosen to optimise the method as it assigns equal weighting to both FP and FN errors.

### Comparison of DendroBLAST against other inference methods

In order to compare DendroBLAST to existing tree inference methods the performance was evaluated on the entire set of 308 simulated protein sequence alignments obtained from [28] excluding the 50 randomly sampled alignments used in the training set above. This final dataset containing 258 simulated protein sequence alignments was specifically chosen so that a direct comparison to multiple other inference methods on an identical dataset could be provided. The resulting trees from other inference methods were also obtained from [28]. To provide a bootstrapped distance method for comparison an additional set of bootstrapped-neighbour-joining tree inferences was performed using QuickTree [8]. All tree inference methods were evaluated on 5 different measures: the Robinson-Foulds distance [32], the number of false positive bipartitions recovered, the number of false negative bipartitions recovered, the precision and the recall. These measures are defined for use in phylogenetic analyses here as:
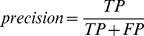
(10)

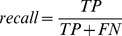
(11)where the true-positives (TP) are the set of bipartitions present in both the reference tree and the inferred tree. The results of each score metric for each inference method were compared to those produced by DendroBLAST using a paired t-test.

### Introduction of alignment error

As the simulated multiple sequence alignments do not contain alignment induced error, an additional test was performed to introduce realistic alignment errors encountered in real multiple sequence alignments. Each of the 308 simulated alignments were parsed to remove all gap characters and realigned using two different methods. 1) MAFFT FFT-NS-1 [33] a fast, accurate and commonly used method for aligning large numbers of sequences. 2) MAFFT L-INS-i, [33] one of the most accurate methods for multiple sequence alignment currently available [3]. To maintain consistency across experiments these realigned sequences were subject to tree inference using a selection of the methods (with identical parameters) described in [28].

### Introduction of alignment trimming

Due negative effects which can be incurred by the inclusion of gap characters and mis-aligned data on phylogenetic inference a common approach is to discarded “gappy” information. Popular methods such as GBLOCKS [34] have been developed to automate this process and thereby reduce the amount of possibly mis-aligned data from multiple sequence alignments. To provide a further test of DendroBLAST against other phylogenetic inference methods we used GBLOCKS to remove columns from the realigned alignments above. Each of the 308 simulated alignments was subject to realignment as above (both L-INS-I and FFT-NS-1) and then parsed using GBLOCKS with options configured for conservative data selection (less data removed than default GBLOCKS settings). The minimum length of an aligned block was set to 5 amino acids, with the number of allowed gap positions set to 50% of the number of sequences and the minimum number of flank positions also set to 50% of the number of sequences.

### Inference of supertrees

To demonstrate the utility of DendroBLAST for tackling real-world datasets, we took an existing dataset of 3537 discrete orthologue groups found in the Archaea [35]. We inferred a DendroBLAST tree for each of the orthologue groups containing 4 or more sequences (n = 1688) and used all of the resulting trees to construct a supertree using two independent quartet supertree methods [36], [37]. The resultant supertree was compared to the phylogenetic tree inferred from the concatenated protein sequences alignments using multiple inference methods [35].

## Results

### A novel method for robust clustering of protein sequences based on BLAST score

**Figure 1:**
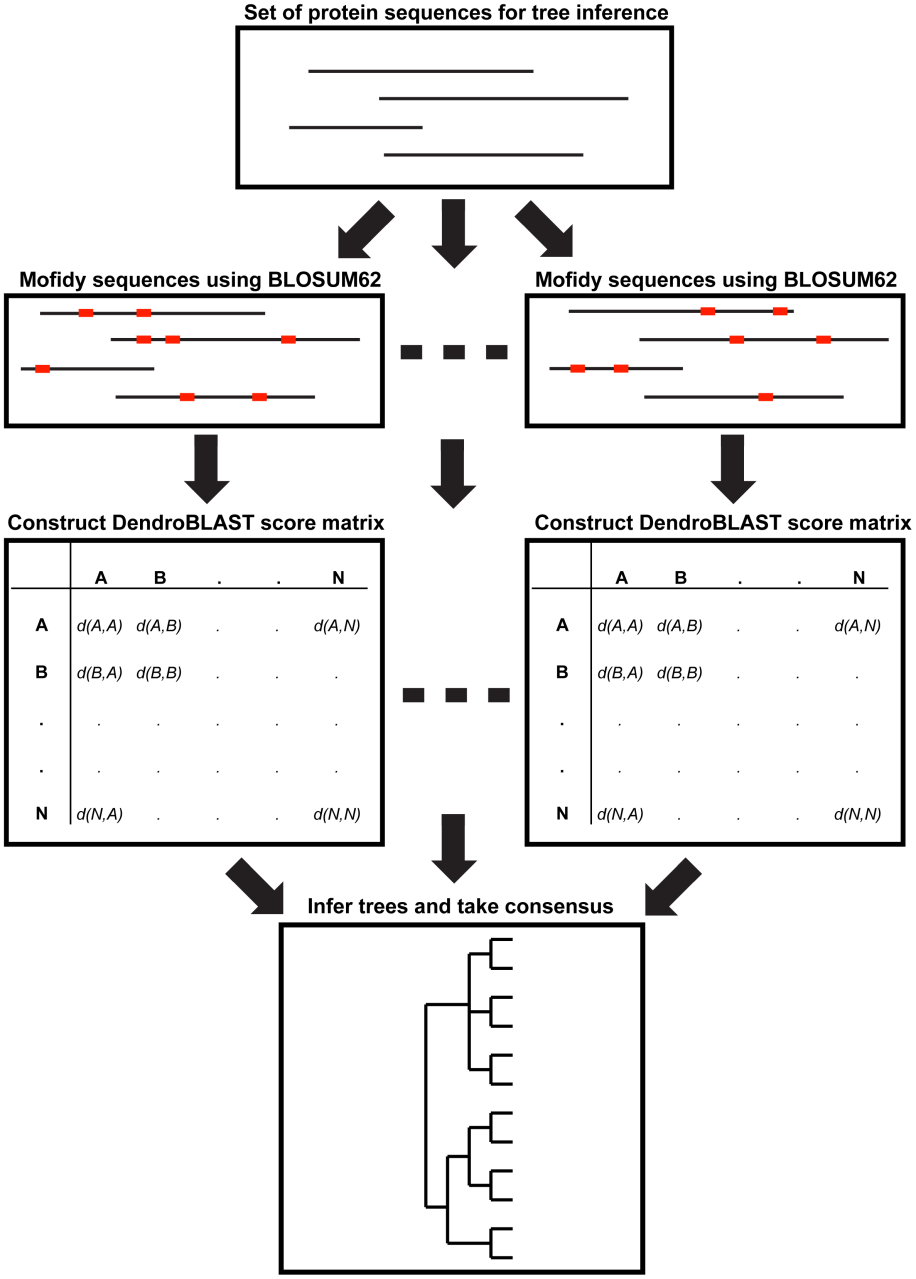
A flow diagram describing the DendroBLAST tree inference procedure. The procedure takes a set of protein sequences and creates 100 copies of this set each with a unique set or randomly introduced sequence changes. Each set of sequences is subject to tree inference and a consensus tree is inferred from these sets. The red bars in the sequences indicate the randomly introduced sequence changes.

We developed a novel method for constructing consensus phylogenetic trees from protein sequences in the absence of multiple sequence alignments. In brief, the method uses minimum evolution clustering of transformed BLAST similarity scores to infer a hierarchical tree of un-aligned protein sequences. A simple model of sequence evolution is then employed to improve the accuracy of the inferred trees by identifying and removing weakly supported bipartitions from the tree. The work flow of the tree inference method is described in Figure 1.

### Accuracy of topological inference in the absence of multiple sequence alignment error

To determine the accuracy of DendroBLAST phylogenetic trees were inferred for each of the 308 simulated protein sequence families present in [28], [29]. Phylogenetic trees have already been produced using several different inference methods for this dataset [28]. These methods comprise RAxML [10], PhyML [38], FastTree [29], FastMe [9], Parsimony (as executed by RAxML [10]) and Neighbour-Joining [8]. Hence, this represents an ideal set on which the performance of DendroBLAST can be directly compared to existing maximum likelihood and distance based methods. All of the above trees were then compared to the reference trees using the dendropy python module [31]. All inference methods were evaluated on the results of 5 score metrics. 1) The number of bipartitions present in the inferred tree but not the reference tree (false positive bipartitions); 2) The number of bipartitions present in the reference tree but not the inferred tree (false negative bipartitions). 3) The Robinson Foulds distance (the sum of false positive and false negative bipartitions). 4) The precision (see methods) and 5) the recall (see methods).

In the above test DendroBLAST generally compares well to other commonly used methods of tree inference (Table 1). DendroBLAST achieves good precision and Robinson-Foulds distance scores (Table 1). However, DendroBLAST fails to recover many bipartitions which are recovered by maximum likelihood methods leading to a poor recall rate (Table 1). Taken together the performance of DendroBLAST is thus better than any of the tested distance methods but is generally out-performed by all of the maximum likelihood methods. In the above test, on average each tree inference using DendroBLAST took 21±3 mins, this includes the time taken to infer 100 trees from DendroBLAST distance matrices and compute the consensus tree. This speed compares well to inferring a single maximum likelihood tree using MAFFT and RAxML where alignment and tree inference took 73±30 mins.

**Table 1:** A comparison of DendroBLAST with other tree inference methods on simulated multiple sequence alignments.

A) Method of tree inference	RFd		FP		FN		Precision		Recall	
PhyML 4G SPR (SH 50) (3)	46.2	a	12.2	a	34.0	a	0.943	a	0.863	a
RAxML (3)	46.8	a	23.4	a	23.4	a	0.906	a	0.906	a
PhyML 4G SPR (3)	49.4	a	24.7	b	24.7	a	0.900	a	0.900	a
PhyML 4G (SH50) (3)	57.7	a	17.5	a	40.2	a	0.918	a	0.838	a
FastTree (SH50) (3)	60.4	a	23.1	a	37.2	a	0.898	a	0.850	a
PhyML 4G (3)	63.4	a	31.7	b	31.7	a	0.872	b	0.872	a
FastTree (3)	64.6	a	32.2	b	32.3	a	0.870	b	0.870	a
PhyML 1G (SH50) (3)	69.1	a	24.7	b	44.4	a	0.888	a	0.821	a
PhyML 1G (3)	75.1	a	37.5	c	37.5	a	0.849	b	0.849	a
DendroBLAST (1)	95.1		27.6		67.5		0.867		0.732	
FastME SPR (2)	96.5	b	48.3	c	48.3	a	0.805	c	0.805	a
FastME (2)	101.2	c	50.6	c	50.6	a	0.796	c	0.796	a
QuickTree log cor. (2)	119.4	c	59.7	c	59.7	a	0.759	c	0.759	a
QuickTree (con50) (2)	142.6	c	67.5	c	75.0	c	0.717	c	0.697	c
QuickTree (2)	149.9	c	74.9	c	74.9	c	0.698	c	0.698	c

B) Method of matrix generation	RFd		FP		FN		Precision		Recall	
Log corrected distances (2)	101.2	a	50.6	a	50.6	a	0.796	a	0.796	a
DendroBLAST distances (1)	122.2		61.1		61.1		0.754		0.754	
Uncorrected distances (2)	122.4	b	61.2	b	61.2	b	0.753	b	0.753	b
Maximum likelihood distances (2)	123.3	b	60.6	b	62.6	b	0.754	b	0.751	b

A) Comparison of DendroBLAST with several alignment based tree inference methods. B) Comparison of tree inference using FastME with distance matrices derived using different methods. In both cases (1) indicates that the tree inference method is distance-based and alignment-free, (2) indicates method is distance-based and alignment-dependent and (3) indicates alignment-dependent. RFd is the Robinson-Foulds distance. FP is the number of false positive bipartitions. FN is the number of false negative bipartitions. StdE is one standard error of the mean. Con 50 indicates that a majority-rule consensus tree was used to evaluate inference performance. SH 50 indicates that only bipartitions supported by an SH test scoring greater than 0.5 were used for evaluating the inference performance. 4G indicates that 4 gamma rate categories were used. SPR is subtree pruning regrafting. “a” indicates performance is significantly better than DendroBLAST, “b” indicates that performance is not significantly different to DendroBLAST and “c” indicates that performance is significantly worse than DendroBLAST. Performance evaluated by paired t-test at p≤0.001. Methods are ordered according to their Robinson-Foulds distance score.

### Comparison of DendroBLAST distance matrices to distance matrices generated by other means

As DendroBLAST produces a distance matrix which is converted to a phylogenetic tree using the minimum evolution principle implemented by FastMe [9] we sought to compare the performance of the DendroBLAST distance measures against established distance measures using the same tree inference method. Here we took the test set of simulated sequence alignments above and computed distance measures using three commonly used methods 1) Uncorrected distance measures 2) Log corrected distance measures computed using FastTree [28] and 3) maximum likelihood distance measures computed using RAxML [10]. These distance matrices were used to infer trees and the accuracy of these trees was interrogated as before. Here all methods except DendroBLAST compute distances using the perfect simulated sequence alignment in this scenario both uncorrected and maximum likelihood distance measures are not significantly different to DendroBLAST distance measures (Table 1).

### Accuracy of topological inference in the presence of multiple sequence alignment error

**Figure 2:**
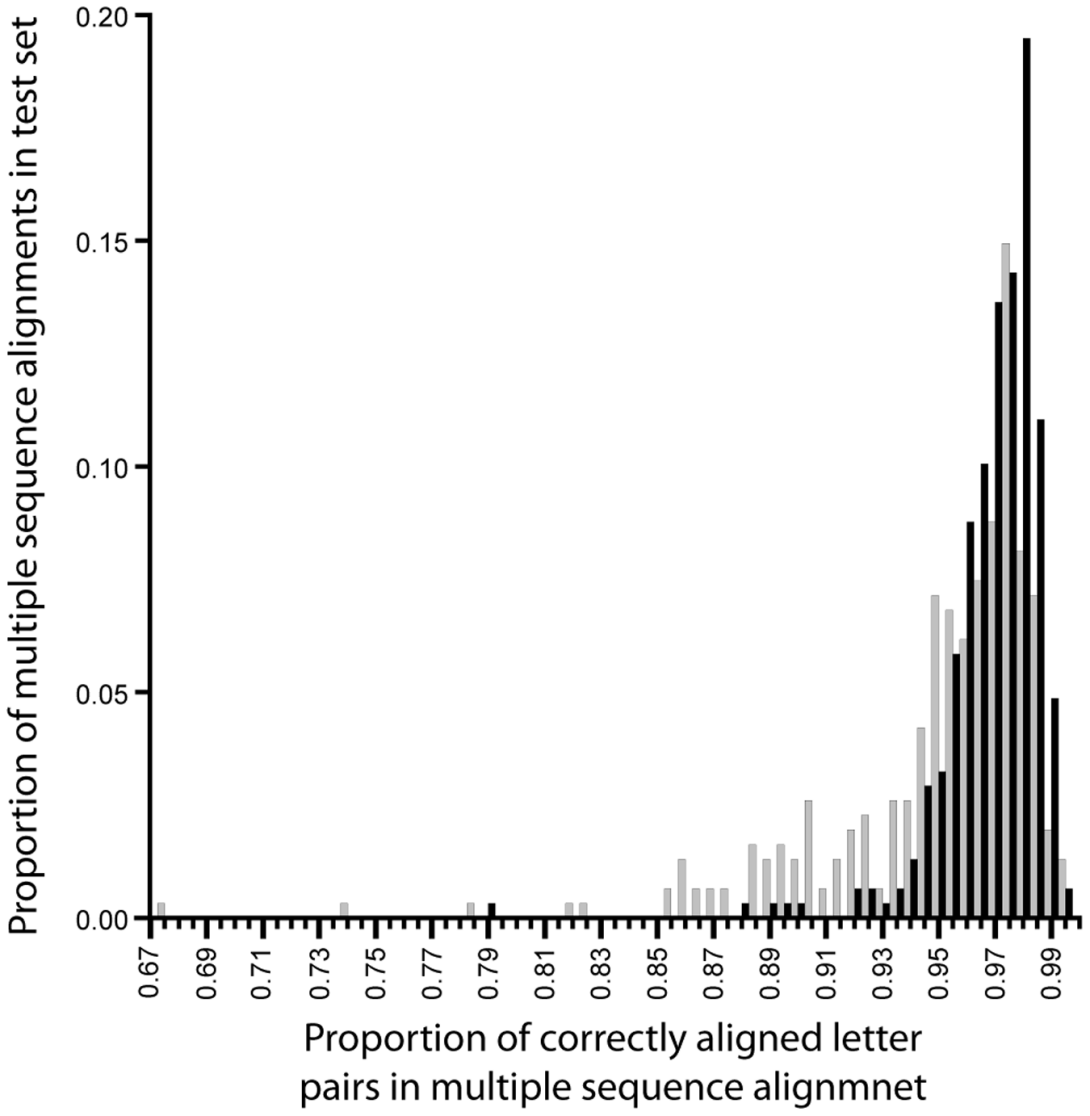
Comparison of different multiple sequence alignment methods on simulated sequence data. Black bars depict results from realignment with L-INS-i method (mean = 0.976). Grey bars depict results from realignment FFT-NS-1 method (mean = 0.952). N = 308.

In the case of the above tests, DendroBLAST was compared to other inference methods each of which used the simulated multiple sequence alignments. The simulated multiple sequence alignments on which these methods were tested do not contain any alignment induced error. In real world situations, the true multiple sequence alignment is not known and hence an additional test of the above inference methods was performed on re-aligned data to introduce realistic errors which occur by the production of a multiple sequence alignment. This presents a more realistic comparison of the performance of DendroBLAST to that of other inference methods. To do this each of the 308 simulated alignments were parsed to remove all gap characters and realigned using two different methods. 1) MAFFT FFT-NS-1 [33] a fast method for aligning large numbers of sequences. 2) MAFFT L-INS-i, [33] one of the most accurate methods for multiple sequence alignment currently available [3]. To evaluate the performance of the selected alignment methods the resulting alignments were compared to the simulated alignments using the Q-score program [39]. For each multiple sequence alignment, the proportion of correctly aligned letter pairs was evaluated. The mean proportion of correctly aligned letter pairs was 0.952 (Standard deviation = 0.039) and 0.976 (Standard deviation = 0.019) for the FFT-NS-1 and L-INS-i methods respectively (Figure 2).

As expected, the introduction of alignment error increased the number of false positive and false negative errors of all alignment-based inference methods (Table 2). As before, DendroBLAST was the most accurate distance based inference method, significantly outperforming both neighbour-joining and minimum evolution (Table 2). However, even considering alignment error, DendroBLAST trees are not as accurate as those inferred using maximum likelihood. For the alignment method which produced least errors (L-INS-i), DendroBLAST achieves a level of precision equivalent to a FastTree or (PhyML with 4 gamma categories) inference. For the alignment method that produced higher error rate (FFT-NS-1), DendroBLAST achieves a precision that is significantly better than FastTree, PhyML (1 or 4 gamma categories) and not significantly different to that of RAxML. This means that in realistic scenarios where moderate levels of multiple sequence alignment error are present, the bipartitions recovered by DendroBLAST are as likely to be correct as those recovered by many maximum likelihood methods. However, as before all maximum likelihood methods achieve a recall score which is better than DendroBLAST. Thus overall DendroBLAST trees are less accurate than maximum likelihood trees.

**Table 2:** A comparison of the performance of different tree inference methods following realignment of simulated sequences.

Realignmnet FFT-NS-1	RFd		FP		FN		Precision		Recall	
PhyML 4G SPR (SH 50) (3)	58.3	a	19.3	a	39.0	a	0.912	a	0.843	a
RAxML (3)	61.2	a	30.6	b	30.6	a	0.877	b	0.877	a
PhyML 4G SPR (3)	63.5	a	31.8	b	31.8	a	0.872	b	0.872	a
PhyML 4G (SH50) (3)	75.4	a	26.7	b	48.7	a	0.877	b	0.804	a
FastTree (SH50) (3)	77.8	a	31.6	b	46.2	a	0.861	b	0.814	a
PhyML 1G (SH50) (3)	82.2	a	30.8	b	51.5	a	0.861	b	0.792	a
FastTree (3)	82.6	a	41.3	c	41.4	a	0.833	c	0.833	a
PhyML 4G (3)	83.4	a	41.7	c	41.7	a	0.832	c	0.832	a
PhyML 1G (3)	89.2	a	44.6	c	44.6	a	0.820	c	0.820	a
DendroBLAST (1)	95.1		27.6		67.5		0.867		0.732	
FastME SPR (2)	112.4	c	56.2	c	56.2	a	0.773	c	0.773	a
FastME (2)	116.1	c	58.1	c	58.1	a	0.766	c	0.766	a
QuickTree log cor. (2)	119.4	c	59.7	c	59.7	a	0.759	c	0.759	a
QuickTree (con50) (2)	149.3	c	70.5	c	78.8	c	0.702	c	0.682	c
QuickTree (2)	157.5	c	78.8	c	78.8	c	0.682	c	0.682	c

Realignment L-INS-i	RFd		FP		FN		Precision		Recall	
PhyML 4G SPR (SH 50) (3)	49.6	a	15.1	a	34.5	a	0.931	a	0.861	a
RAxML (3)	51.8	a	25.9	b	25.9	a	0.895	a	0.895	a
PhyML 4G SPR (3)	54.0	a	27.0	b	27.0	a	0.891	a	0.891	a
PhyML 4G (SH50) (3)	57.7	a	17.5	a	40.2	a	0.918	a	0.838	a
PhyML 4G (3)	63.4	a	31.7	b	31.7	a	0.872	b	0.872	a
FastTree (SH50) (3)	66.2	a	26.0	b	40.2	a	0.886	a	0.838	a
FastTree (3)	70.4	a	35.1	c	35.2	a	0.858	b	0.858	a
PhyML 1G (SH50) (3)	74.1	a	26.9	b	47.2	a	0.878	a	0.810	a
PhyML 1G (3)	80.0	a	40.0	c	40.0	a	0.839	a	0.839	a
DendroBLAST (1)	95.1		27.6		67.5		0.867		0.732	
FastME SPR (2)	102.9	c	51.5	c	51.5	a	0.792	c	0.792	c
FastME (2)	106.9	c	53.4	c	53.4	a	0.785	c	0.785	c
QuickTree log cor. (2)	119.4	c	59.7	c	59.7	a	0.759	c	0.759	a
QuickTree (con50) (2)	145.0	c	68.5	c	76.4	c	0.712	c	0.692	c
QuickTree (2)	152.6	c	76.3	c	76.3	c	0.692	c	0.692	c

Please refer to the legend for table 1 for explanations of abbreviations.

### Introduction of alignment trimming

In the case of the above experiment, DendroBLAST was compared to other inference methods using simulated multiple sequence alignments with addition of alignment induced error. It is common in phylogenetic analysis for alignments to be subject to trimming before use. Trimming removes positions which are suspected to contain mis-aligned sequence and hence could lead to phylogenetic error. However, trimming also reduces the amount of data available to make the inference and hence can negatively affect phylogenetic inference through data reduction. Here a commonly used package for alignment trimming GBLOCKS [34] was used to trim the re-aligned multiple sequence alignments using a conservative (less data removed) setting. In all cases trimming the re-aligned multiple sequence alignments resulted in reduction of inference performance (Table 3) using alignment based methods. This effect was more pronounced on the alignments which contained higher error rates (Table 3). This result agrees with similar findings which suggest that removing data using methods like GBLOCKS does not always improve the accuracy of phylogenetic inference [40], [41].

**Table 3:** A comparison of the performance of different tree inference methods following trimming of realigned simulated sequences.

GBLOCKS trimmed FFT-NS-1	RFd		FP		FN		Precision		Recall	
PhyML 4G SPR (SH 50) (3)	68.4	a	21.8	a	46.5	a	0.896	a	0.813	a
RAxML (3)	74.1	a	37.0	c	37.0	a	0.851	b	0.851	a
PhyML 4G SPR (3)	76.3	a	38.1	c	38.1	a	0.847	c	0.847	a
PhyML 4G (SH50) (3)	82.1	a	27.4	b	54.7	a	0.868	b	0.780	a
FastTree (SH50) (3)	86.9	a	35.4	c	51.5	a	0.843	b	0.793	a
PhyML 4G (3)	87.1	a	30.9	c	56.2	a	0.855	c	0.775	a
FastTree (3)	92.2	a	46.0	c	46.2	a	0.814	c	0.814	a
PhyML 1G (SH50) (3)	93.1	a	46.6	c	46.5	a	0.814	c	0.811	a
DendroBLAST (1)	95.1		27.6		67.5		0.867		0.732	
PhyML 1G (3)	96.5	b	48.2	c	48.2	a	0.806	c	0.806	a
FastME SPR (2)	119.5	c	59.8	c	59.8	a	0.759	c	0.759	a
FastME (2)	123.7	c	61.9	c	61.9	a	0.751	c	0.751	a
QuickTree log cor. (2)	124.0	c	62.0	c	62.0	a	0.748	c	0.748	c
QuickTree (con50) (2)	128.3	c	28.8	b	99.5	c	0.834	c	0.599	c
QuickTree (2)	161.6	c	80.9	c	80.9	c	0.674	c	0.674	c

GBLOCKS trimmed L-INS-i	RFd		FP		FN		Precision		Recall	
PhyML 4G SPR (SH 50) (3)	56.4	a	17.1	a	39.3	a	0.920	a	0.842	a
RAxML (3)	61.0	a	30.5	b	30.5	a	0.876	b	0.876	a
PhyML 4G (SH50) (3)	61.8	a	18.1	a	43.8	a	0.912	c	0.822	c
PhyML 4G SPR (3)	62.7	a	31.4	c	31.4	a	0.873	b	0.873	a
PhyML 4G (3)	68.8	a	34.4	c	34.4	a	0.859	c	0.859	b
FastTree (SH50) (3)	74.7	a	29.7	a	45.0	a	0.869	b	0.819	a
PhyML 1G (SH50) (3)	77.1	c	27.0	c	50.2	a	0.875	c	0.798	b
FastTree (3)	79.5	a	39.6	c	39.8	a	0.840	b	0.840	a
PhyML 1G (3)	84.7	a	42.4	c	42.4	a	0.830	c	0.829	a
DendroBLAST (1)	95.1		27.6		67.5		0.867		0.732	
FastME SPR (2)	108.0	c	54.0	c	54.0	a	0.782	c	0.782	a
FastME (2)	112.1	c	55.9	c	55.9	a	0.775	c	0.775	a
QuickTree log cor. (2)	122.6	c	61.3	c	61.3	a	0.752	c	0.752	b
QuickTree (con50) (2)	124.2	c	27.9	b	96.3	c	0.843	c	0.612	c
QuickTree (2)	154.8	c	77.4	c	77.4	c	0.688	c	0.688	c

Please refer to the legend for table 1 for explanations of abbreviations.

### Application to real-world data

**Figure 3:**
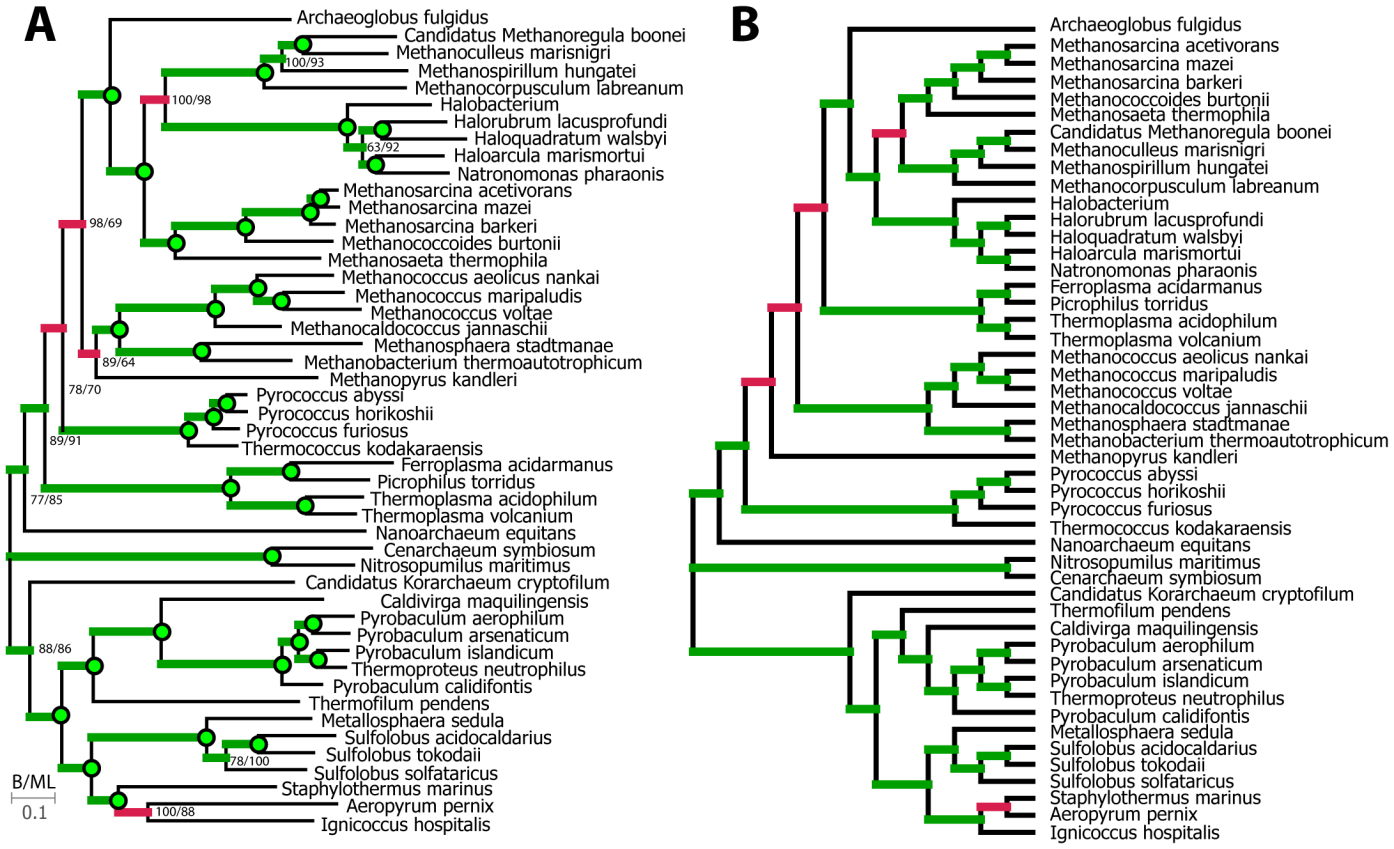
Comparison of DendroBLAST derived supertree with concatenated protein sequence phylogeny. A) Phylogenetic tree produced from concatenated multiple sequence alignment using maximum likelihood and Bayesian methods. Green circles indicate 100% support under both methods. Numbers at nodes indicate percent support from bootstrapped analyses. Green branches indicate bipartitions found in tree B, red branches indicate bipartitions absent from tree B. B) Supertree constructed from quartets derived from DendroBLAST trees. Green branches indicate bipartitions found in tree A, red branches indicate bipartitions not found in tree A.

To provide an independent test of the DendroBLAST method and demonstrate its utility for analysis of real-world sequence data, we selected a large dataset which has been shown by multiple inference methods (comprising Bayesian and Maximum likelihood analyses of concatenated gene trees as well as quartet supertrees from individual maximum likelihood gene trees) to support the same tree topology [35]. This dataset comprises 3537 orthologous groups differentially distributed across the Archaea. For each of the orthologous groups containing 4 or more sequences (n = 1688), a DendroBLAST tree and used the inferred trees to construct a supertree using two independent quartet supertree methods [36], [37]. The two resulting DendroBLAST derived supertrees were identical and showed at total of 5 missing bipartitions (Figure 3A, recall = 0.89) and 5 additional bipartitions (Figure 3B, precision = 0.89) when compared to the previously published tree (Figure 3A). Interestingly, none of the bipartitions missed by DendroBLAST obtain 100% support by Bayesian and maximum likelihood methods (Figure 3A).

## Discussion

We present a novel BLAST-based tree inference algorithm that achieves good levels of precision in the absence of multiple sequence alignments. In all tests performed on realistic simulated sequence data, DendroBLAST outperformed all of the commonly used distance based methods achieving levels of precision comparable to that of maximum likelihood methods. However, DendroBLAST recall rate is comparatively poor rendering it less accurate in comparison to tree inference using maximum likelihood methods from good quality multiple sequence alignments. We therefore do not propose DendroBLAST as a replacement for high quality phylogenetic inference methods but rather as a platform for improving and informing multiple aspects of bioinformatic analysis.

One such application of this method may be in constructing guide trees for informing multiple sequence alignment. As DendroBLAST bypasses a multiple sequence alignment step and produces good quality approximate phylogenetic trees, use of DendroBLAST may prevent propagation of multiple sequence alignment error incurred from poor quality guide trees. This would be particularly suitable for difficult to align sequence families and in situations where the accuracy of the multiple sequence alignment is in question. Improvement in the accuracy of the guide tree in these scenarios is likely to lead to improvements in the accuracy of the subsequent alignment and tree inference. A strictly bifurcating guide tree is also produced by DendroBLAST and available using the online implementation at http://www.dendroblast.com/.

While the bipartitions that are recovered by DendroBLAST are likely to be correct, DendroBLAST does have a low recall rate in comparison to other maximum likelihood methods meaning than many bipartitions are missed. One use for high-precision low-recall phylogenetic trees is in construction of supertrees. We demonstrate the utility of DendroBLAST for this task by reconstructing a supertree of the Archaea. This supertree reconstruction closely matched previous Bayesian and maximum likelihood analyses with a precision and recall of 0.89 (taking these previous analyses as reference). Hence, we propose that this method may be useful for production of phylogenetic trees for use in supertree reconstruction or in providing approximate start trees for subsequent optimisation.

## Supporting Information

File S1 **Perl implementation of DendroBLAST method.**
(PL) Click here for additional data file.

Figure S1 **Optimising the scale of the Gamma distribution.** A) Plot of the effect of varying the Gamma distribution scale factor on the false positive partitions, false negative partitions and Robinson-Foulds distance. Value at 0 is estimated from 100 replicates with no amino acid replacement. Black line indicates the fitted polynomial model, the local optimum for Gamma scale value is 1.9644. Error bars indicate 1 standard error of the mean (n = 50). B) The frequency of the amino acid changes for a 100 replicate DendroBLAST inference using the optimal Gamma scale parameter. For example, a value of 0 indicates that the amino acid was not changed. A value of 1 indicates that the amino acid was changed to an amino acid which has a score of 1 less than the score for not changing in the BLOSUM62 substitution matrix. C) The distribution of score values in the remapped BLOSUM62 substitution matrix. D) Comparison of pairwise distances computed by DendroBLAST and by PhyML using 4 gamma rate categories.
(PDF)

## References

[1] Golubchik T, Wise MJ, Easteal S, Jermiin LS (2007) Mind the gaps: evidence of bias in estimates of multiple sequence alignments. Mol Biol Evol 24: 2433–2442. 17709332 10.1093/molbev/msm176

[2] Edgar RC (2010) Quality measures for protein alignment benchmarks. Nucleic Acids Res 38: 2145–2153. 20047958 10.1093/nar/gkp1196 PMC2853116

[3] Thompson JD, Linard B, Lecompte O, Poch O (2011) A comprehensive benchmark study of multiple sequence alignment methods: current challenges and future perspectives. PLoS One 6: e18093. 21483869 10.1371/journal.pone.0018093 PMC3069049

[4] Ogdenw TH, Rosenberg MS (2006) Multiple sequence alignment accuracy and phylogenetic inference. Syst Biol 55: 314–328. 16611602 10.1080/10635150500541730

[5] Hartmann S, Vision TJ (2008) Using ESTs for phylogenomics: can one accurately infer a phylogenetic tree from a gappy alignment? BMC Evol Biol 8: 95. 18366758 10.1186/1471-2148-8-95 PMC2359737

[6] Cantarel BL, Morrison HG, Pearson W (2006) Exploring the relationship between sequence similarity and accurate phylogenetic trees. Mol Biol Evol 23: 2090–2100. 16891377 10.1093/molbev/msl080

[7] Dwivedi B, Gadagkar SR (2009) Phylogenetic inference under varying proportions of indel-induced alignment gaps. BMC Evol Biol 9: 211. 19698168 10.1186/1471-2148-9-211 PMC2746219

[8] Howe K, Bateman A, Durbin R (2002) QuickTree: building huge Neighbour-Joining trees of protein sequences. Bioinformatics 18: 1546–1547. 12424131 10.1093/bioinformatics/18.11.1546

[9] Desper R, Gascuel O (2002) Fast and accurate phylogeny reconstruction algorithms based on the minimum-evolution principle. J Comput Biol 9: 687–705. 12487758 10.1089/106652702761034136

[10] Stamatakis A (2006) RAxML-VI-HPC: maximum likelihood-based phylogenetic analyses with thousands of taxa and mixed models. Bioinformatics 22: 2688–2690. 16928733 10.1093/bioinformatics/btl446

[11] Huelsenbeck JP, Ronquist F (2001) MRBAYES: Bayesian inference of phylogenetic trees. Bioinformatics 17: 754–755. 11524383 10.1093/bioinformatics/17.8.754

[12] Novak A, Miklos I, Lyngso R, Hein J (2008) StatAlign: an extendable software package for joint Bayesian estimation of alignments and evolutionary trees. Bioinformatics 24: 2403–2404. 18753153 10.1093/bioinformatics/btn457

[13] Liu K, Raghavan S, Nelesen S, Linder CR, Warnow T (2009) Rapid and accurate large-scale coestimation of sequence alignments and phylogenetic trees. Science 324: 1561–1564. 19541996 10.1126/science.1171243

[14] Suchard MA, Redelings BD (2006) BAli-Phy: simultaneous Bayesian inference of alignment and phylogeny. Bioinformatics 22: 2047–2048. 16679334 10.1093/bioinformatics/btl175

[15] Hagopian R, Davidson JR, Datta RS, Samad B, Jarvis GR, et al (2010) SATCHMO-JS: a webserver for simultaneous protein multiple sequence alignment and phylogenetic tree construction. Nucleic Acids Res 38: W29–34. 20430824 10.1093/nar/gkq298 PMC2896197

[16] Yona G, Linial N, Linial M (2000) ProtoMap: automatic classification of protein sequences and hierarchy of protein families. Nucleic Acids Res 28: 49–55. 10592179 10.1093/nar/28.1.49 PMC102438

[17] Kriventseva EV, Servant F, Apweiler R (2003) Improvements to CluSTr: the database of SWISS-PROT+TrEMBL protein clusters. Nucleic Acids Res 31: 388–389. 12520029 10.1093/nar/gkg035 PMC165482

[18] Kelil A, Wang S, Brzezinski R, Fleury A (2007) CLUSS: clustering of protein sequences based on a new similarity measure. BMC Bioinformatics 8: 286. 17683581 10.1186/1471-2105-8-286 PMC1976428

[19] Enright AJ, Van Dongen S, Ouzounis CA (2002) An efficient algorithm for large-scale detection of protein families. Nucleic Acids Res 30: 1575–1584. 11917018 10.1093/nar/30.7.1575 PMC101833

[20] Jothi R, Zotenko E, Tasneem A, Przytycka TM (2006) COCO-CL: hierarchical clustering of homology relations based on evolutionary correlations. Bioinformatics 22: 779–788. 16434444 10.1093/bioinformatics/btl009 PMC1620014

[21] Liu X, Wan L, Li J, Reinert G, Waterman MS, et al (2011) New powerful statistics for alignment-free sequence comparison under a pattern transfer model. J Theor Biol 284: 106–116. 21723298 10.1016/j.jtbi.2011.06.020 PMC3146591

[22] Reinert G, Chew D, Sun F, Waterman MS (2009) Alignment-free sequence comparison (I): statistics and power. J Comput Biol 16: 1615–1634. 20001252 10.1089/cmb.2009.0198 PMC2818754

[23] Vinga S, Almeida J (2003) Alignment-free sequence comparison-a review. Bioinformatics 19: 513–523. 12611807 10.1093/bioinformatics/btg005

[24] Altschul SF, Madden TL, Schaffer AA, Zhang J, Zhang Z, et al (1997) Gapped BLAST and PSI-BLAST: a new generation of protein database search programs. Nucleic Acids Res 25: 3389–3402. 9254694 10.1093/nar/25.17.3389 PMC146917

[25] Lake JA, Servin JA, Herbold CW, Skophammer RG (2008) Evidence for a new root of the tree of life. Syst Biol 57: 835–843. 19085327 10.1080/10635150802555933

[26] Walter R (1976) Principles of Mathematical Analysis. New York: McGraw-Hill.

[27] Loewenstein Y, Portugaly E, Fromer M, Linial M (2008) Efficient algorithms for accurate hierarchical clustering of huge datasets: tackling the entire protein space. Bioinformatics 24: i41–49. 18586742 10.1093/bioinformatics/btn174 PMC2718652

[28] Price MN, Dehal PS, Arkin AP (2009) FastTree: computing large minimum evolution trees with profiles instead of a distance matrix. Mol Biol Evol 26: 1641–1650. 19377059 10.1093/molbev/msp077 PMC2693737

[29] Price MN, Dehal PS, Arkin AP (2010) FastTree 2–approximately maximum-likelihood trees for large alignments. PLoS One 5: e9490. 20224823 10.1371/journal.pone.0009490 PMC2835736

[30] Tatusov RL, Fedorova ND, Jackson JD, Jacobs AR, Kiryutin B, et al (2003) The COG database: an updated version includes eukaryotes. BMC Bioinformatics 4: 41. 12969510 10.1186/1471-2105-4-41 PMC222959

[31] Sukumaran J, Holder MT (2010) DendroPy: a Python library for phylogenetic computing. Bioinformatics 26: 1569–1571. 20421198 10.1093/bioinformatics/btq228

[32] Robinson D, Foulds L (1981) Comparison of phylogenetic trees. Mathematical Biosciences 53: 131–147.

[33] Katoh K, Kuma K, Miyata T, Toh H (2005) Improvement in the accuracy of multiple sequence alignment program MAFFT. Genome Inform 16: 22–33. 16362903

[34] Talavera G, Castresana J (2007) Improvement of phylogenies after removing divergent and ambiguously aligned blocks from protein sequence alignments. Syst Biol 56: 564–577. 17654362 10.1080/10635150701472164

[35] Kelly S (2010) Archaeal phylogenomics provides evidence in support of a methanogenic origin of the Archaea and a thaumarchaeal origin for the eukaryotes. Philos Trans R Soc Lond B Biol Sci 10.1098/rspb.2010.1427 PMC3049024 20880885

[36] Piaggio-Talice R, Burleigh G, Eulenstein O (2004) Quartet Supertrees:. In: Bininda-Edmonds ORP, editor. Phylogenetic Supertrees: Combining Information to Reveal the Tree of Life: Springer. pp. 173–191.

[37] Holland B, Conner G, Huber K, Moulton V (2007) Imputing supertrees and supernetworks from quartets. Syst Biol 56: 57–67. 17366137 10.1080/10635150601167013

[38] Guindon S, Gascuel O (2003) A simple, fast, and accurate algorithm to estimate large phylogenies by maximum likelihood. Syst Biol 52: 696–704. 14530136 10.1080/10635150390235520

[39] Edgar RC (2004) MUSCLE: multiple sequence alignment with high accuracy and high throughput. Nucleic Acids Res 32: 1792–1797. 15034147 10.1093/nar/gkh340 PMC390337

[40] Dessimoz C, Gil M (2010) Phylogenetic assessment of alignments reveals neglected tree signal in gaps. Genome Biol 11: R37. 20370897 10.1186/gb-2010-11-4-r37 PMC2884540

[41] Jordan G, Goldman N (2012) The effects of alignment error and alignment filtering on the sitewise detection of positive selection. Mol Biol Evol 29: 1125–1139. 22049066 10.1093/molbev/msr272

