# Supplementary material for: DendroBLAST: Approximate Phylogenetic Trees in the Absence of Multiple Sequence Alignments
Source: PLoS One. 2013 Mar 15;8(3):e58537. doi: 10.1371/journal.pone.0058537 (PMC3598851; doi:10.1371/journal.pone.0058537)
Supplement: Figure S1 — Optimising the scale of the Gamma distribution. A) Plot of the effect of varying the Gamma distribution scale factor on the false positive partitions, false negative partitions and Robinson-Foulds distance. Value at 0 is estimated from 100 replicates with no amino acid replacement. Black line indicates the fitted polynomial model, the local optimum for Gamma scale value is 1.9644. Error bars indicate 1 standard error of the mean (n = 50). B) The frequency of the amino acid changes for a 100 replicate DendroBLAST inference using the optimal Gamma scale parameter. For example, a value of 0 indicates that the amino acid was not changed. A value of 1 indicates that the amino acid was changed to an amino acid which has a score of 1 less than the score for not changing in the BLOSUM62 substitution matrix. C) The distribution of score values in the remapped BLOSUM62 substitution matrix. D) Comparison of pairwise distances computed by DendroBLAST and by PhyML using 4 gamma rate categories. (PDF) [file pone.0058537.s002.pdf]

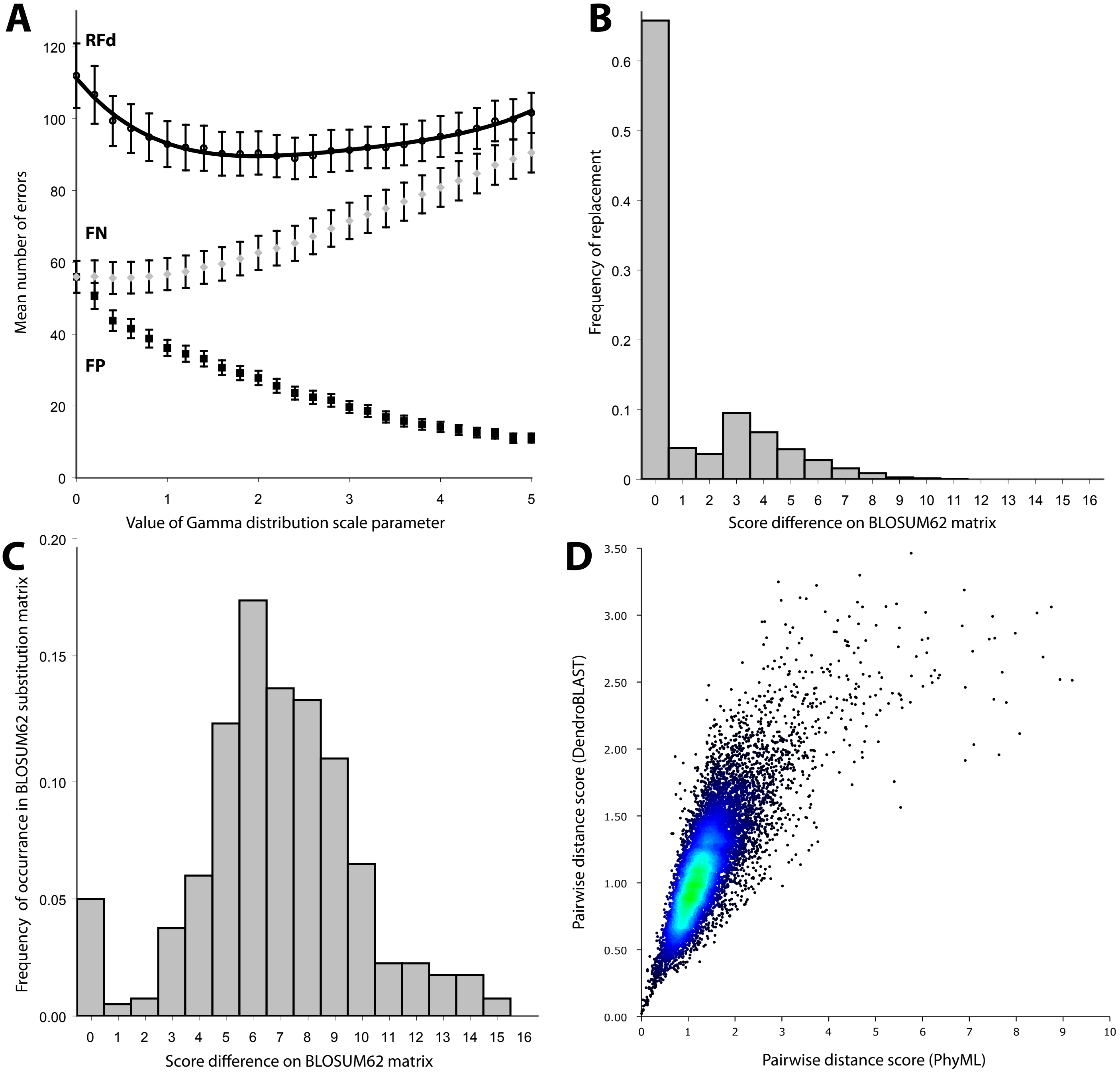

Figure S2: Optimising the scale of the Gamma distribution. **A)** Plot of the effect of varying the Gamma distribution scale factor on the false positive partitions, false negative partitions and Robinson-Foulds distance. Value at 0 is estimated from 100 replicates with no amino acid replacement. Black line indicates the fitted polynomial model, the local optimum for Gamma scale value is 1.9644. Error bars indicate 1 standard error of the mean. **B)** The frequency of the amino acid changes for a 100 replicate DendroBLAST inference using the optimal Gamma scale parameter. For example, a value of 0 indicates that the amino acid was not changed. A value of 1 indicates that the amino acid was changed to an amino acid which has a score of 1 less than the score for not changing in the BLOSUM62 substitution matrix. **C)** The distribution of score values in the remapped BLOSUM62 substitution matrix. D) Comparison of pairwise distances computed by DendroBLAST and by PhyML using 4 gamma rate categories.
